# Advancing personalized, predictive, and preventive medicine in bladder cancer: a multi-omics and machine learning approach for novel prognostic modeling, immune profiling, and therapeutic target discovery

**DOI:** 10.3389/fimmu.2025.1572034

**Published:** 2025-04-22

**Authors:** Han Yan, Xinyu Ji, Bohan Li

**Affiliations:** ^1^ Department of Pain Medicine, The First Hospital of China Medical University, Liaoning, Shenyang, China; ^2^ Department of Thoracic Surgery, The First Hospital of China Medical University, Liaoning, Shenyang, China; ^3^ Department of Urinary Surgery, The First Hospital of China Medical University, Liaoning, Shenyang, China

**Keywords:** bladder cancer, immunogenic cell death, machine learning, multi-omics integration, immunotherapy, prognosis signature

## Abstract

**Objective:**

This study aimed to identify and analyze immunogenic cell death (ICD)-related multi-omics features in bladder cancer (BLCA) using single-cell RNA sequencing (scRNA-seq) and bulk RNA-seq data. By integrating these datasets, we sought to construct a prognostic signature (ICDRS) and explore its clinical and biological implications, including its association with immune cell infiltration, tumor microenvironment (TME), and drug sensitivity.

**Methods:**

Publicly available datasets from TCGA and GEO, including scRNA-seq (GSE222315, 9 samples) and bulk RNA-seq (TCGA-BLCA, 403 samples; GSE13507, 160 samples), were analyzed. Single-cell data were processed using Seurat, and ICD scores were calculated using single-sample gene set enrichment analysis (ssGSEA). Weighted gene co-expression network analysis (WGCNA) identified ICD-related modules, and machine learning algorithms (Lasso, Ridge, CoxBoost) were employed to construct the ICDRS. Survival analysis, immune infiltration, pathway enrichment, and drug sensitivity were evaluated to validate the model.

**Results:**

The ICDRS, based on eight key genes (IL32, AHNAK, ANXA5, FN1, GSN, CNN3, FXYD3, CTSS), effectively stratified BLCA patients into high- and low-risk groups with significant differences in overall survival (OS, P < 0.001). High ICDRS scores were associated with immune-suppressive TME, including increased infiltration of T cells CD4 memory resting (P = 0.02) and macrophages M0/M1/M2 (P = 0.01). Pathway enrichment revealed correlations with cholesterol homeostasis, epithelial-mesenchymal transition (EMT), and KRAS signaling. Drug sensitivity analysis showed high-risk groups were resistant to Cisplatin (P = 0.003), Mitomycin C (P = 0.01), and Paclitaxel (P = 0.004), with IC50 values significantly higher than low-risk groups.

**Conclusion:**

The ICDRS serves as a robust prognostic biomarker for BLCA, offering insights into tumor immune evasion mechanisms and potential therapeutic targets. Its integration with clinical features enhances personalized treatment strategies, highlighting the importance of ICD in BLCA immunotherapy and precision medicine. The model’s predictive accuracy and biological relevance were validated across multiple datasets, underscoring its potential for clinical application.

## 1 Introduction

Over the past few decades, the advent of personalized, predictive, and preventive medicine (PPPM/3PM) has significantly transformed the landscape of cancer treatment and research (1). Among urological malignancies, bladder cancer (BLCA) stands as the second most prevalent, with an estimated 550,000 new cases and approximately 200,000 deaths reported annually worldwide (2). The management of BLCA continues to be a critical focus in urological oncology. Platinum-based chemotherapy remains the cornerstone of perioperative and advanced BLCA treatment. More recently, the introduction of immune checkpoint inhibitors (ICIs) has expanded therapeutic options, particularly for first-line and platinum-resistant cases (3). Despite these advancements, the proportion of patients experiencing long-term remission through immunotherapy remains limited. The overall response rate of ICIs in BLCA is no more than 24% (4). To address this, future research must prioritize the exploration of novel immunotherapeutic strategies, the optimization of therapeutic sequencing and combinations, the precise selection of therapies tailored to individual patients, and the identification of new molecular targets.

Within the realm of BLCA immunotherapy, the investigation of immunogenic cell death (ICD) within the tumor microenvironment (TME) has emerged as a pivotal research avenue. ICD is characterized by the release of damage-associated molecular patterns (DAMPs), which activate the immune system to combat tumors. This process involves the recruitment of antigen-presenting cells (APCs) to damaged or infected cells, leading to the presentation of captured antigens via major histocompatibility complex proteins to primary T cells. Consequently, ICD enhances the antigenicity of tumor cells and promotes robust anti-tumor immune responses (5–7). As a novel biomarker, ICD holds considerable promise. ICD has been extensively studied in various cancer types, demonstrating its central role in antitumor immunity. Conventional chemotherapeutics like anthracyclines, oxaliplatin, and taxanes have been shown to induce ICD in multiple malignancies, enhancing immunogenicity and correlating with improved patient outcomes (8). However, its clinical applications, such as prognostic stratification and the prediction of responses to immunotherapy and chemotherapy, remain underexplored (9). A comprehensive understanding of ICD at the molecular level, coupled with advanced analytical techniques, is essential for its accurate identification and effective utilization.

Recognizing the potential of ICD in BLCA treatment, the systematic identification of its associated multi-omics features is imperative for the development of innovative therapeutic strategies. Furthermore, the application of diverse machine learning frameworks to analyze these complex datasets not only enhances the robustness of the research but also uncovers the intricate interactions and integration mechanisms among different data types. This approach is instrumental in identifying key biomarkers and molecular pathways associated with BLCA (10).

In light of these considerations, this study aims to employ a range of machine learning computational frameworks to systematically identify and analyze ICD-related multi-omics features in BLCA. By integrating genomics, transcriptomics, and proteomics data, we seek to elucidate key molecules and pathways that influence BLCA immune responses. Additionally, we aim to provide new targets for precision therapy and construct risk models using follow-up data. This research not only offers a novel perspective on understanding the immunoregulatory mechanisms of BLCA and advancing its precision treatment but also underscores the significant potential of machine learning in the analysis of complex diseases within the 3PM framework.

## 2 Materials and methods

### 2.1 Data source

In this study, we utilized publicly available genomic data from two primary sources: The Cancer Genome Atlas (TCGA) and the Gene Expression Omnibus (GEO) database. From the TCGA database (https://portal.gdc.cancer.gov/), we obtained gene expression profiles and corresponding survival data for 403 BLCA samples, providing a robust dataset for analysis (11). Additionally, we accessed two BLCA-specific datasets from the GEO database (https://www.ncbi.nlm.nih.gov/geo/): GSE222315, which includes single-cell RNA sequencing (scRNA-seq) data from 9 bladder cancer samples, and GSE13507, which contains tissue-based RNA sequencing data and survival information from 160 bladder cancer samples (12).

For the TCGA dataset, we extracted gene expression data in Transcripts Per Million (TPM) format from STAR count data. The data were normalized using a log2(TPM+1) transformation to stabilize variance and approximate a normal distribution. To ensure the reliability of downstream analyses, we retained only those samples with both RNA sequencing data and complete clinical information. This approach allowed us to integrate diverse data types while maintaining methodological consistency and analytical rigor.

### 2.2 Single-cell RNA sequencing analysis

#### 2.2.1 Data preprocessing

The scRNA-seq data were processed using the Seurat package (version 5.1.0) in R (version 4.3.1) (13). Quality control was performed to remove low-quality cells, retaining those with between 200 and 6,000 detected genes and mitochondrial gene content below 5%. Red blood cell contamination was assessed using hemoglobin genes (e.g., *HBA1*, *HBB*), and samples with more than 1% hemoglobin gene expression were excluded. The data were normalized using the LogNormalize method with a scale factor of 10,000, and highly variable genes were identified using the FindVariableFeatures function, retaining the top 3,000 genes for downstream analysis.

To address batch effects, the Harmony algorithm was applied, ensuring robust integration across samples. Harmony was specifically chosen for our batch correction strategy because of its demonstrated effectiveness in single-cell data integration. The algorithm works by iteratively clustering cells and adjusting their positions in the reduced dimensional space to align shared cell populations across datasets, while preserving biologically meaningful variations. Unlike other integration methods that may overcorrect and remove important biological signals, Harmony maintains cell type-specific transcriptional signatures while minimizing technical variation. This balanced approach was critical for our analysis, as we needed to integrate multiple scRNA-seq datasets while preserving the subtle transcriptional differences that characterize distinct immune cell states and their interactions within the tumor microenvironment. Additionally, Harmony’s computational efficiency allowed us to process our large-scale integrated dataset without compromising resolution or accuracy, making it particularly well-suited for our multi-source data integration approach. Principal component analysis (PCA) was conducted, and the top 30 principal components were selected for dimensionality reduction and clustering. t-distributed Stochastic Neighbor Embedding (tSNE) (14) was employed with parameters perplexity=30 and max_iter=1000 to visualize the data, and clusters were identified using the FindNeighbors and FindClusters functions with a resolution of 0.8. Cell types were annotated based on well-established marker genes, including *CD3D/CD3E* for T cells, *CD19/MS4A1* for B cells, *COL1A1/COL1A2* for fibroblasts, *PECAM1* for endothelial cells, *CD163/CD68/CD14* for macrophages, and *APC/GSTP1/CDKN2A/DAPK1* for cancer cells. Marker genes for each cluster were identified using FindAllMarkers with parameters logfc.threshold=0.35 and min.pct=0.35.

#### 2.2.2 Immunogenic cell death analysis

To investigate the role of ICD in the tumor microenvironment, a curated list of ICD-related genes was used to compute an ICD score for each cell using single-sample gene set enrichment analysis (ssGSEA) (15). Cells were stratified into high and low ICD score groups using the median score as the cutoff. Differential expression analysis between these groups was performed using the FindAllMarkers function, with a log2 fold change threshold of 0.35 and a minimum detection rate of 35%.

Pathway enrichment analysis was conducted using the clusterProfiler package, and significant pathways were identified using gene set enrichment analysis (GSEA) on the Hallmark gene sets. The results were visualized using dot plots and enrichment plots to highlight pathways associated with high ICD scores. UMAP plots were generated to visualize the distribution of ICD scores across cell types, and violin plots were used to compare ICD scores between annotated cell types. Statistical significance was defined as p < 0.05.

#### 2.2.3 Cell-cell communication analysis

The CellChat package was employed to infer cell-cell communication networks based on ligand-receptor interactions (16). The human CellChatDB database was used to identify overexpressed ligand-receptor pairs, and communication probabilities were computed using the computeCommunProb function. Significant interactions were filtered (min.cells = 10), and pathway-level communication networks were aggregated using the computeCommunProbPathway function.

Key signaling pathways, such as MIF, ITGB2, MK, and APP, were visualized using circle plots and heatmaps to illustrate their strength and specificity across cell types. Chord diagrams were generated to depict interactions between high-risk tumor cells and other cell types, providing insights into the role of cell-cell communication in the tumor microenvironment.

To further explore the impact of risk-associated genes on cell-cell communication, cells were classified into high- and low-risk groups based on a predefined set of genes (e.g., *IL32*, *AHNAK*, *ANXA5*) using ssGSEA. Differential expression analysis between these groups was performed, and enriched pathways were identified using GSEA. The results were visualized using dot plots and enrichment plots, highlighting pathways associated with high-risk cells. Statistical significance was defined as p < 0.05.

### 2.3 Weighted gene co-expression network analysis

To identify co-expression modules and explore their associations with ICD in BLCA, we performed WGCNA using the R package WGCNA (17). Gene expression data from TCGA-BLCA samples were preprocessed, and ICD-related genes were selected based on a curated gene list. The ssGSEA method was employed to calculate ICD scores for each sample, which were used as traits in the WGCNA analysis.

The expression matrix was filtered to retain genes with high variability, and a soft-thresholding power was determined to construct a scale-free network. Modules of co-expressed genes were identified using hierarchical clustering and dynamic tree cutting. Module-trait relationships were assessed by correlating module eigengenes with ICD scores. The yellow module, which showed the strongest association with ICD, was further analyzed. Gene significance and module membership were calculated to identify key genes within the yellow module.

Functional enrichment analysis of the yellow module genes was performed using the clusterProfiler package to uncover biological processes and pathways associated with ICD. Visualization included dendrograms, heatmaps, and scatterplots to illustrate module-trait relationships and gene significance. Statistical significance was defined as p < 0.05.

### 2.4 Integrating machine learning methods to construct prognostic features

We conducted an intersection analysis between ICD-DEGs and the module genes identified through WGCNA to obtain a set of genes related to immunogenic cell death (ICDRgenes). To construct a robust prognostic model with high predictive accuracy, we randomly divided the TCGA-BLCA dataset into a training set (302 samples) and an internal testing set (101 samples) in a 3:1 ratio, ensuring an even distribution of clinical characteristics between the two groups. Furthermore, the GSE13507 dataset (160 samples) served as an external testing set to ensure the robustness of the model.

During the model construction phase, our study incorporated various machine learning algorithms, including least absolute shrinkage and selection operator (Lasso) (18), stepwise multiple Cox (StepCox) (19), Ridge (20), CoxBoost (21), Survival Support Vector Machine (Survival-SVM) (22), Elastic Net (Enet) (23, 24), Partial Least Squares Regression for Cox Models and Related Techniques (plsRcox) (25, 26), Supervised Principal Components (SuperPC) (27), Random Survival Forests (RSF) (28), and Gradient Boosting Machine (GBM) (29). We arranged 100 combinations of these 10 algorithms across the TCGA-BLCA and GSE13507 datasets, employing a ten-fold cross-validation framework for variable selection and model building.

Ultimately, we selected the algorithm combination that demonstrated the best robust performance and potential for clinical translation based on its performance across the three datasets. This led to the establishment of a final feature set called the immunogenic cell death-related signature (ICDRS), which is used to predict overall survival (OS) in BLCA patients.

### 2.5 Survival analysis and construction of nomograms

Based on the median risk score of the ICDRS, we divided the samples in the TCGA training set, internal testing set, and external testing set into high-risk and low-risk groups. Kaplan-Meier(KM) survival curves were analyzed using the R package survminer (30), and differences in OS between the high-risk and low-risk groups were compared using the log-rank test. Additionally, the timeROC package (31) was used to perform ROC curve analysis to assess the sensitivity and specificity of ICDRS in predicting OS in BLCA patients, with the area under the curve (AUC) reflecting the robustness of the model (32, 33). Further, we stratified the ICDRS scores by clinical characteristics and analyzed the correlation of ICDRS with age, gender, tumor stage, T, M, N classification, and other clinical features.

To enhance the predictive accuracy and prognostic capability of our model, we developed a nomogram (34) that combines ICDRS with clinical features to quantify the expected survival of BLCA patients. Finally, we comprehensively evaluated the precision and accuracy of the nomogram through ROC curves, the concordance index (C-index), and calibration curves. Additionally, we used decision curve analysis (DCA) (35) to assess the clinical net benefit of the nomogram, ensuring its practicality and effectiveness in clinical decision support. These comprehensive assessments helped us validate the clinical application value of the nomogram, ensuring its contribution to the survival prediction of BLCA patients in actual medical settings. Statistical significance was defined as p < 0.05.

### 2.6 Comprehensive analysis of immune characteristics and responses to immune checkpoint inhibitor therapy

To explore the relationship between immune cell infiltration within TME of BLCA and ICDRS, we used the IOBR package (36) to assess ESTIMATE scores, CIBERSORT infiltration estimates, and the infiltration of 28 types of immune cells in BLCA samples from TCGA. Firstly, we employed the CIBERSORT algorithm to quantify the infiltration of 22 immune cell types. CIBERSORT is a computational method based on the principle of linear support vector regression (SVR) and uses a predefined reference gene expression feature matrix (LM22) to deconvolute RNA sequencing data. The LM22 matrix includes characteristic expression profiles of 22 immune cell types, allowing us to accurately estimate the relative abundance of these cell types at the RNA transcription level, facilitating the identification and quantification of cell types (37).

Following the initial analysis, we refined our results on immune cell infiltration using the immune phenotype scoring method for 28 types of immune cells, as published by Charoentong et al. (38). We then utilized the ESTIMATE algorithm to assess the correlation between immune cells and genes in tumor samples. The combined use of these methods not only provided a multidimensional perspective on immune cell infiltration but also helped us to verify and enhance the precision and robustness of the CIBERSORT estimates. This ensured that our study results accurately and deeply elucidated the relationship between immune cell infiltration and survival prognosis in BLCA. Statistical significance was defined as p < 0.05.

### 2.7 Significance of the ICDRS in drug sensitivity

We utilized the Genomics of Drug Sensitivity in Cancer (GDSC) database (https://www.cancerrxgene.org/) to predict the sensitivity of samples from high and low-risk groups to common anticancer drugs. This database is one of the largest public resources in the field of pharmacogenomics, providing rich data on drug sensitivity and related genomic information, which is crucial for identifying potential cancer treatment targets (39). We employed the pRRophetic package to construct a Ridge regression model based on cell lines, using the gene expression profiles and risk scores of ICDRS from BLCA to estimate the half-maximal inhibitory concentration (IC50) of drug samples (40). This method allows us to assess the sensitivity of different ICDRS risk groups (high risk and low risk) to anticancer drugs.

### 2.8 HPA validation

We validated the protein expression of relevant genes in BLCA using the Human Protein Atlas (HPA) database. The HPA database (https://www.proteinatlas.org/) is the most extensive and comprehensive resource on the spatial distribution of proteins in human tissues and cells (41). By integrating advanced transcriptomics and proteomics technologies, the HPA database provides detailed information on protein expression at both RNA and protein levels across various human tissues and organs. This approach allows for a thorough validation of the protein expression of genes of interest in BLCA, providing valuable insights into their biological relevance and potential therapeutic significance.

## 3 Results

**Figure 1:**
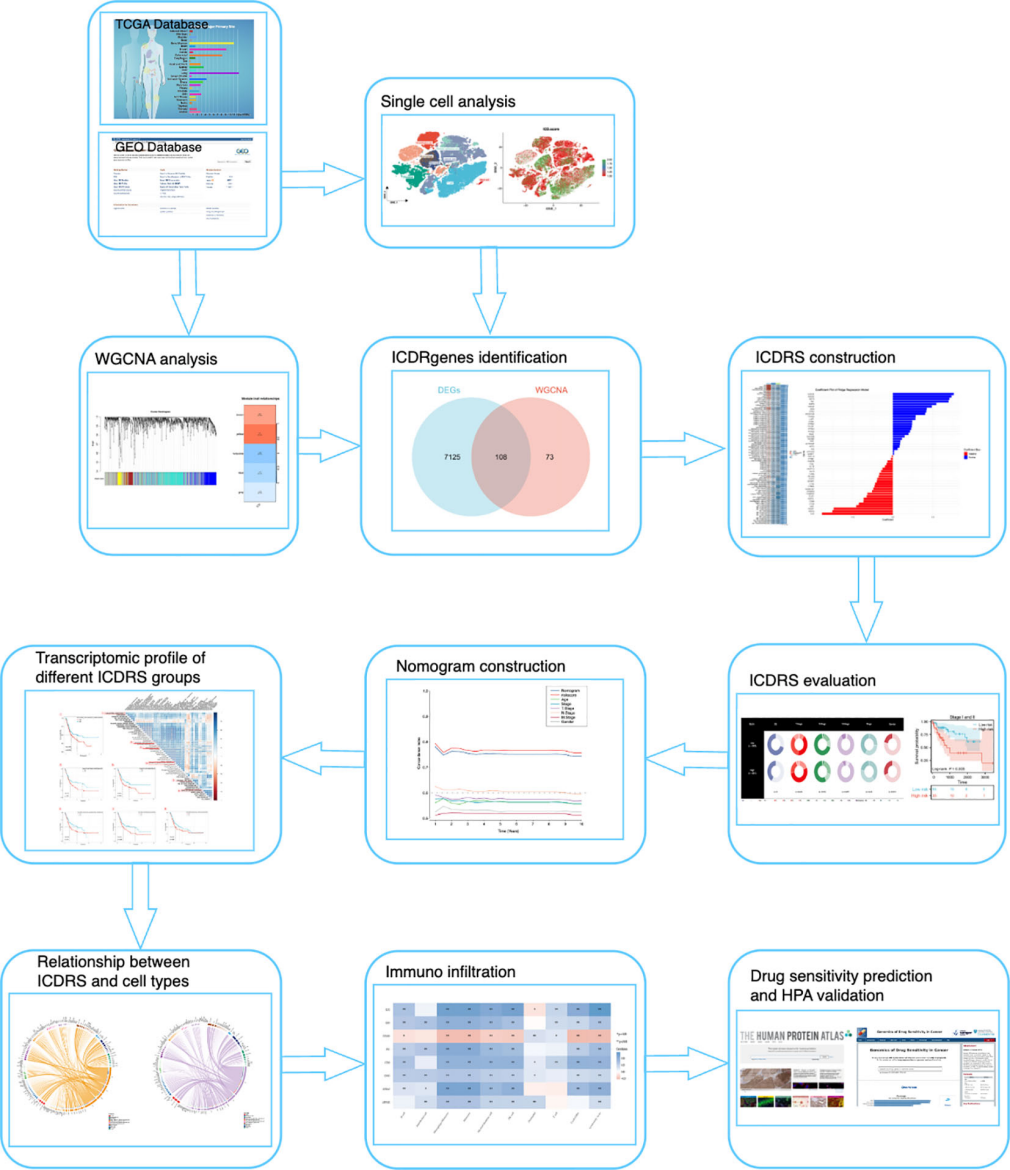
Study flowchart.

All analytical processes are illustrated in the flowchart (**Figure 1**).

### 3.1 ICD features of single-cell transcriptomics

**Figure 2:**
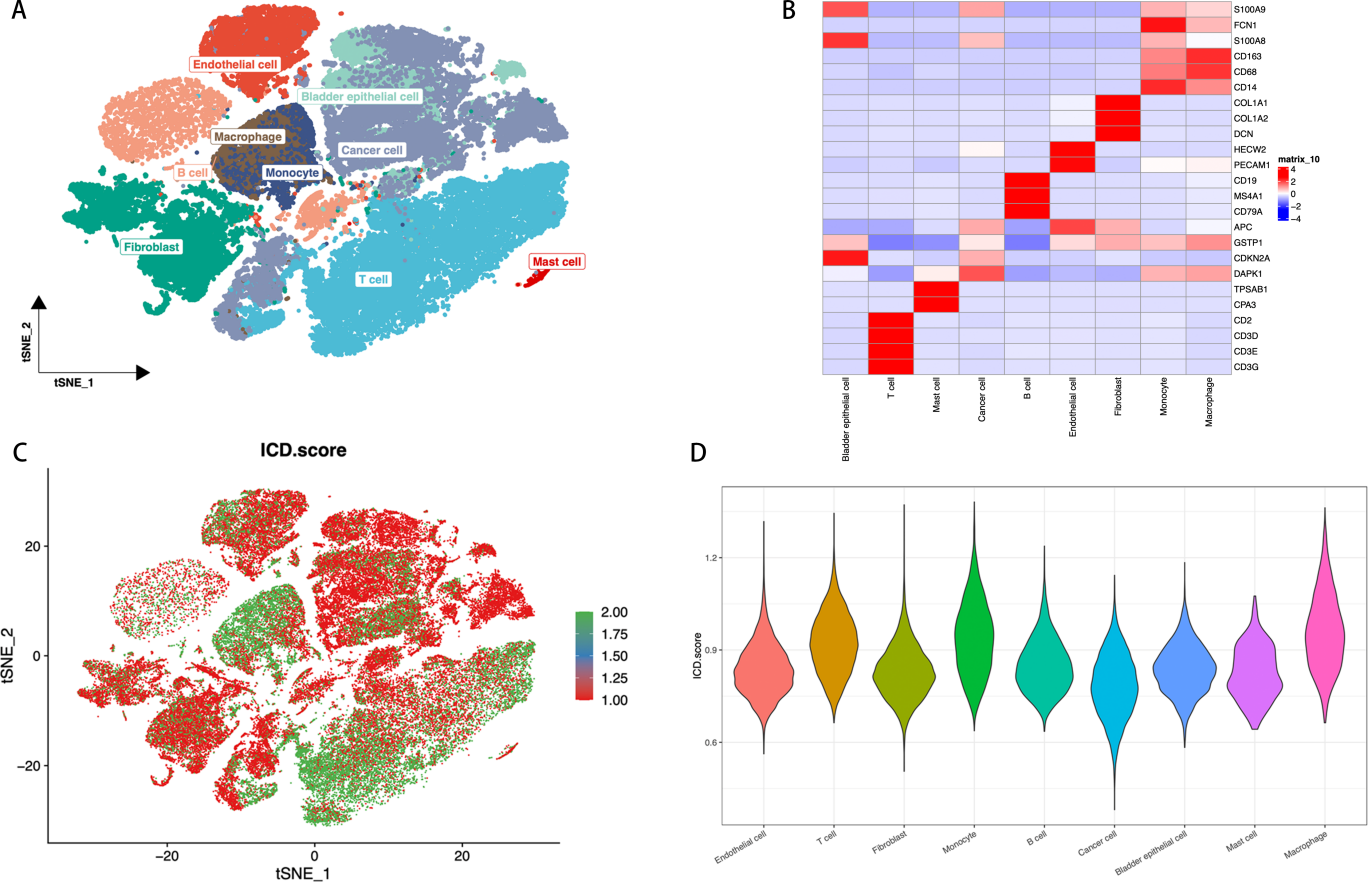
ICD Features in Single-Cell Transcriptomics. **(A)** t-SNE plot showing the cell types identified by marker genes. **(B)** Heatmap displaying the marker genes in each cell cluster, where red and blue respectively indicate high and low gene expression levels. **(C)** The activity score of ICD in each cell. **(D)** The distribution of the ICD scores in different cell types, with the width of each violin plot shape indicating the density and range of ICD scores in the corresponding cell type.

We assessed the single-cell transcriptomic landscape to characterize the features of ICD across different cell types. Utilizing t-SNE technology, we set marker genes according to previous relevant literatures (42, 43), then identified cell types defined by marker genes, annotating cells into 9 major clusters: endothelial cells, bladder epithelial cells, macrophages, monocytes, cancer cells, fibroblasts, mast cells, B cells, and T cells, thereby revealing the distribution patterns of various cell populations (**Figure 2**). In the heatmap, we displayed the expression of marker genes for each cell cluster, such as high expression of CD68 and CD163 in macrophages (**Figure 2**). We further assessed the ICD activity score in each single cell (**Figure 2**). Using a continuous color gradient from green (low ICD score) to red (high ICD score), we observed the distribution of ICD activity among different cell groups. Finally, we presented the distribution of ICD scores across different cell types by violin plots (**Figure 2**). Results indicated that immune cells like macrophages, T cells, and monocytes exhibited higher ICD scores, while non-immune cells such as fibroblasts, endothelial cells, and cancer cells showed lower scores. Based on ICD activity, we categorized cells into high ICD and low ICD groups and identified 7,233 ICD-DEGs between the two groups for further analysis.

### 3.2 Identifying ICDRgenes in bulk RNA sequencing

We utilized the TCGA-BLCA sample dataset and applied the WGCNA method to identify and analyze genes related to the immunogenic cell death-related modules.

**Figure 3:**
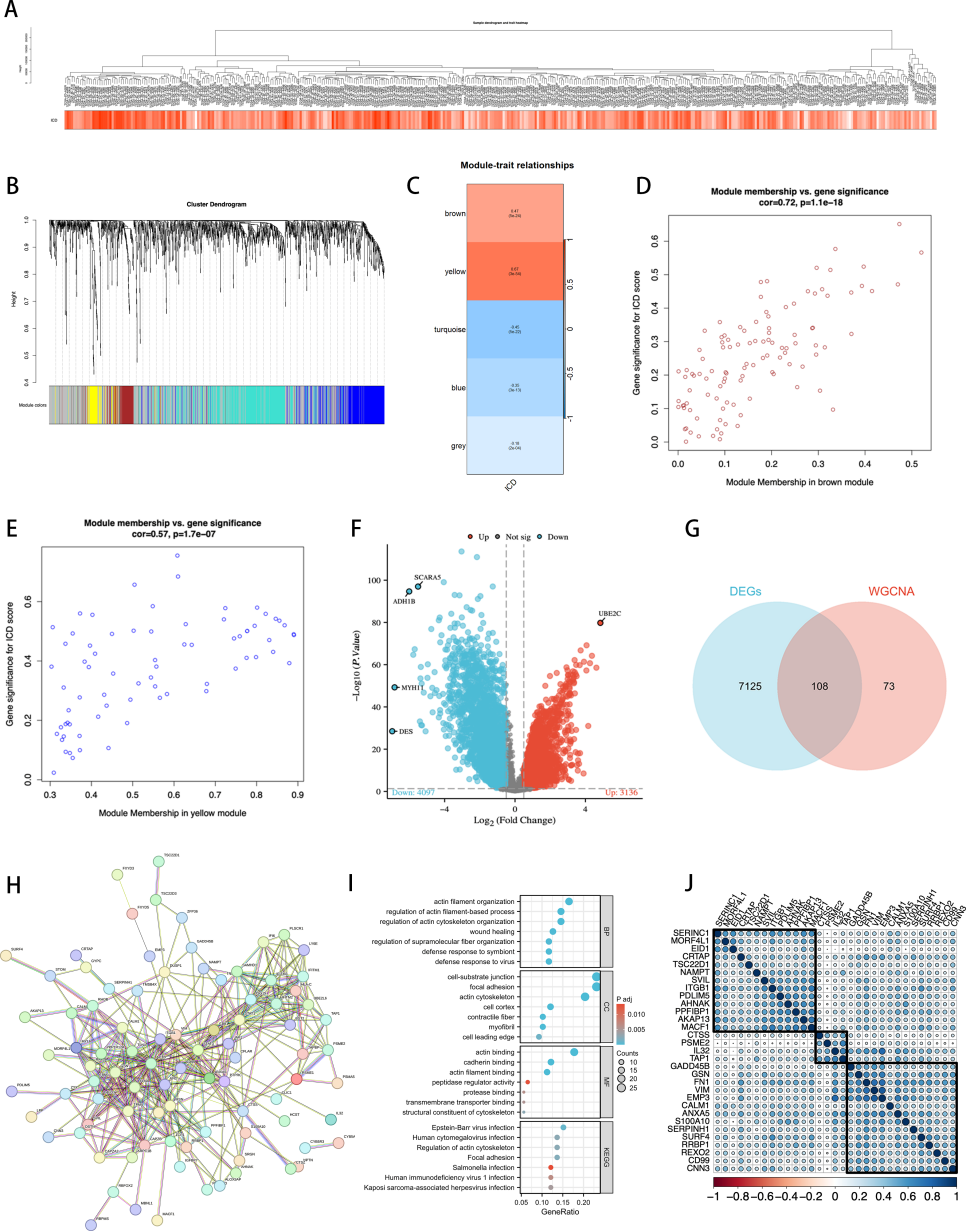
Identification of ICDRgenes. **(A)** Dendrogram showing the hierarchical clustering of TCGA-BLCA samples. The heatmap at the bottom represents the ICD scores of each sample. **(B)** Cluster dendrogram of the WGCNA analysis. **(C)** Module-trait heatmap showing that the brown and yellow modules were closely related to the ICD trait. **(D)** Scatter plot showing the relationship between GS and MM in the brown module. **(E)** The relationship between GS and MM in the yellow module. **(F)** Volcano plot showing differential analysis results between TCGA-BLCA samples and normal samples, with the top 5 most significantly different genes specially marked. **(G)** Venn plot showing the intersecting genes between the two modules and DEGs in bulk RNA-seq. **(H)** PPI Network of 108 ICD-DEGs. **(I)** GO and KEGG Analysis Results. **(J)** After using Cox regression and cross-validation, 108 genes resulted in 31 statistically significant genes related to prognosis, which were then divided into 3 clusters for correlation analysis.

By constructing a hierarchical clustering dendrogram of the samples (**Figure 3**), we displayed the clustering relationships among tumor samples. The heatmap at the bottom shows each sample’s ICD score to illustrate the relative activity of ICD features within the samples. In WGCNA, we constructed a dendrogram of sample clustering (**Figure 3**) and revealed through a module-trait heatmap (**Figure 3**) that the brown and yellow modules are closely associated with ICD traits. In the brown module, the scatterplot of gene significance (GS) and module membership (MM) relationships (**Figure 3**) shows a positive correlation between them. We further displayed the gene expression differences in single-cell tumor samples classified by ICD scores through a volcano plot (**Figure 3**). Additionally, using a Venn diagram (**Figure 3**), we identified 108 intersecting genes between the two modules and bulk RNA sequencing ICD-DEGs, termed ICDRgenes, which are considered to be significantly involved in ICD at both the whole and single-cell transcriptomic levels.

Subsequently, we established a protein-protein interaction network (PPI network) composed of 108 ICDRgenes (**Figure 3**), revealing potential interactions among these genes. We then conducted GO and KEGG enrichment analyses (**Figure 3**) to explore the distribution of these ICDRgenes in BP, CC, and MF, as well as their potential roles in various biological pathways. The results showed that these genes are primarily enriched in pathways related to the regulation of the actin cytoskeleton, which was further confirmed in KEGG analysis.

To further construct and validate the model, we performed a Cox cross-validation with the TCGA gene list and clinical prognosis data, identifying 31 genes with significant statistical significance. After correlation analysis, these genes were further divided into three clusters (**Figure 3**), which were used for subsequent analyses.

### 3.3 Construction of prognostic features based on integrated machine learning

**Figure 4:**
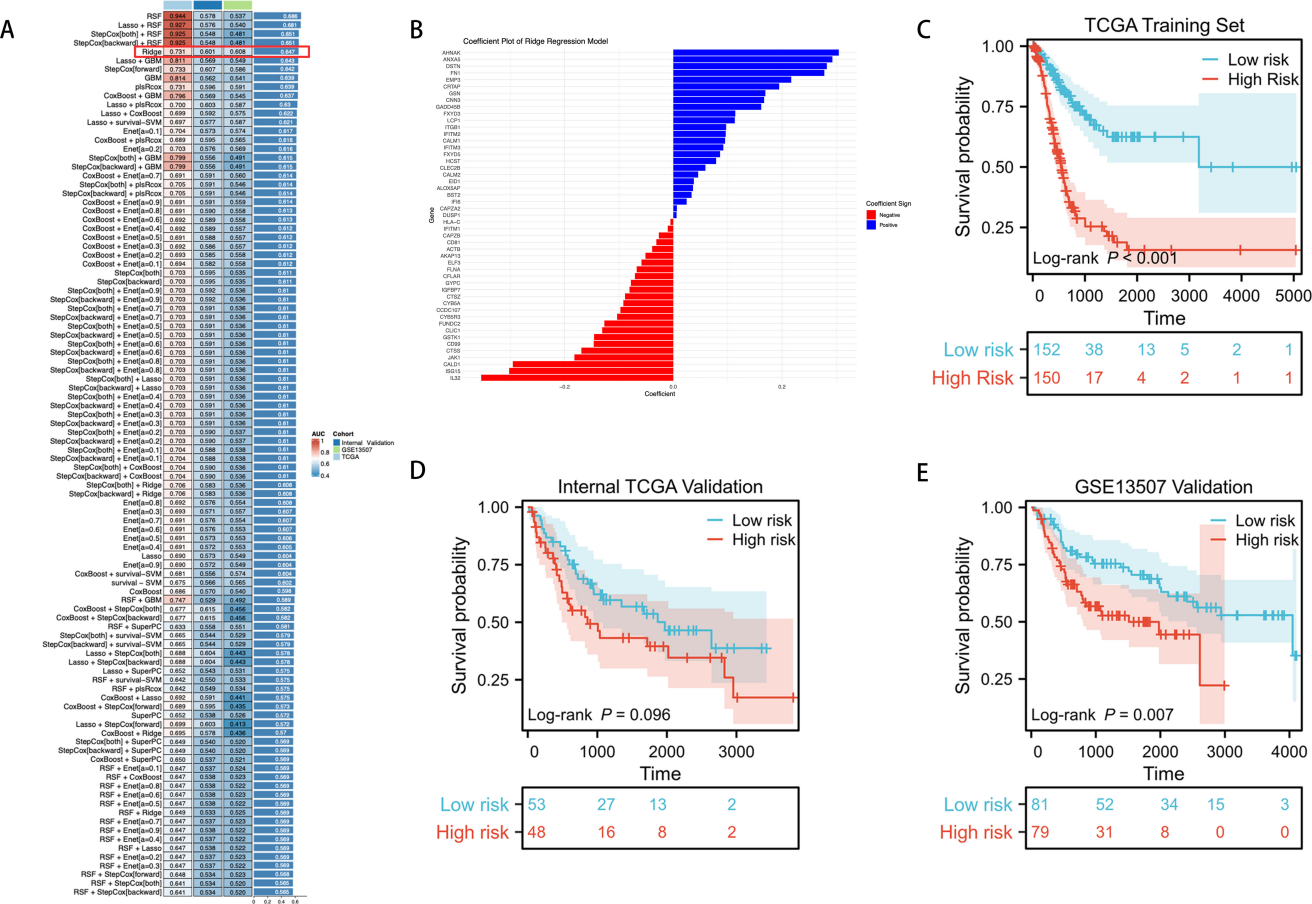
Through a machine learning-based integration process, we developed and validated a consensus ICDRS. **(A)** We evaluated 100 predictive models within a ten-fold cross-validation framework and further calculated the C-index for each model across all validation datasets. **(B)** We visualized the variable weights in the Ridge model, where red indicates negative weights, blue represents positive weights, and the size denotes the absolute value of the weight. **(C–E)** KM curves for OS of ICDRS in the TCGA training set, TCGA internal testing set, and GSE13507 external testing set based on the log-rank test.

We utilized an integrated machine learning approach to develop a consensus ICDRS. Within a ten-fold cross-validation framework, we evaluated 100 different predictive models by assessing the accuracy of each model across all datasets (**Figure 4**). The main criteria for model selection was the best balance between predictive performance and model stability across all datasets, with minimal reduction in C-index values between training and testing sets compared to other algorithms. Considering the overall performance, we selected the Ridge model for constructing the ICDRS and displayed the variable weights within the Ridge model (**Figure 4**). These weights reflect the importance of each variable in the model.

We further validated the prognostic capabilities of the ICDRS using KM curves in three independent datasets. In the TCGA training set, patients stratified into low-risk and high-risk groups based on the ICDRS showed significant differences in survival (P < 0.001) (**Figure 4**). In the internal TCGA testing set, while there was a trend indicating survival differences, the differences did not reach statistical significance (P = 0.096) (**Figure 4**). However, in the external GSE13507 testing set, the high and low scores of the ICDRS demonstrated significant statistical differences in survival (P = 0.007) (**Figure 4**).

### 3.4 Assessing the performance of the ICDRS

**Figure 5:**
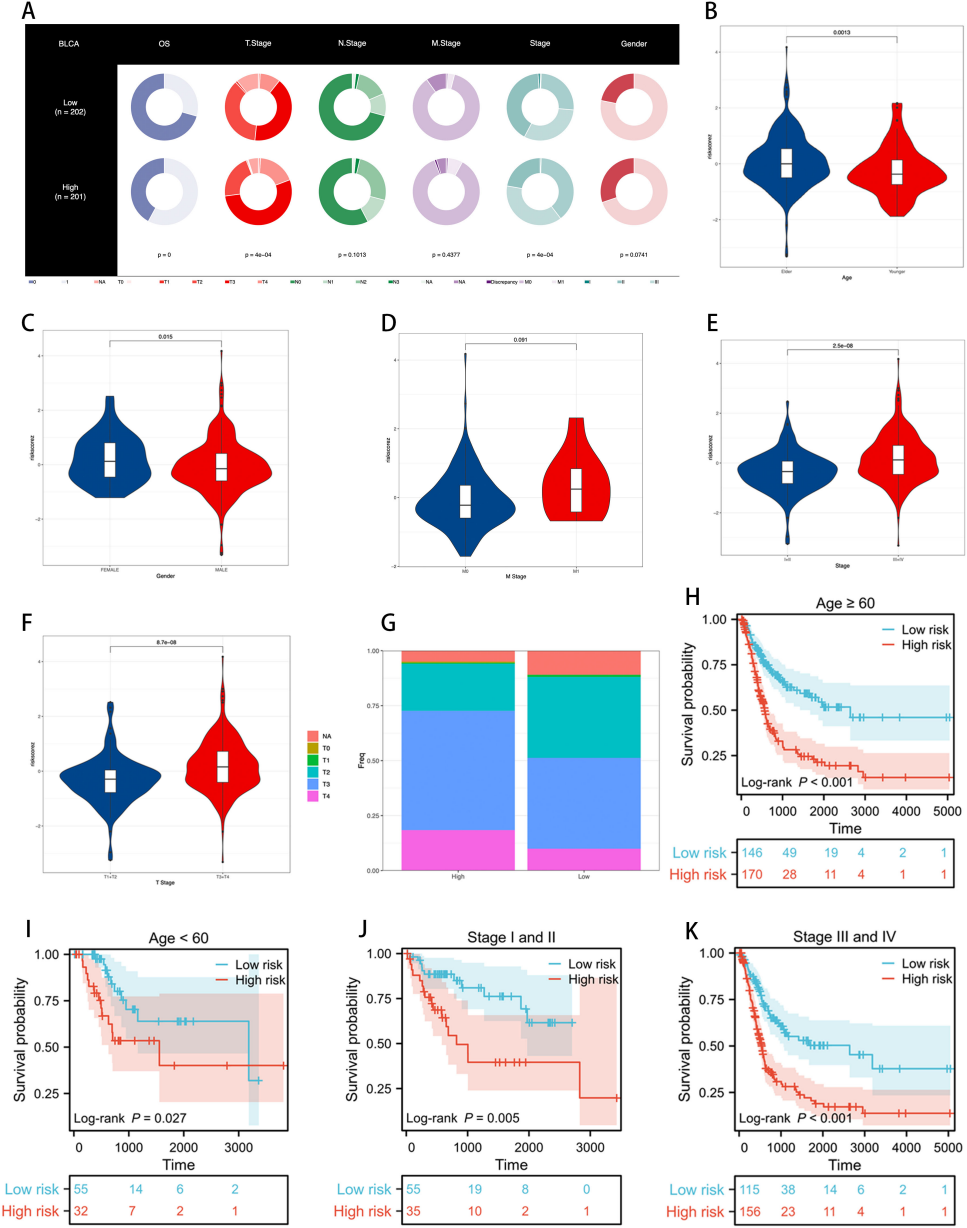
Assessing the performance of the ICDRS. **(A)** Clinical characteristics of ICDRS low-risk and high-risk groups are visualized through the proportions in each ring, illustrating the risk distribution within each subgroup, with p-values used to assess the correlation between ICDRS and these clinical features. **(B–F)** Differences in risk scores between patients grouped by age, gender, M stage, clinical stage, and T stage. **(G)** The proportion of T stage distribution in ICDRS risk subgroups, showing the variation in early versus late stages across different risk levels. **(H–K)** KM curves demonstrating the stable performance of the ICDRS in subgroups, including age and stage.

We assessed the distribution and performance of the ICDRS across different clinical characteristic subgroups of BLCA patients. **Figure 5** shows the distribution of ICDRS low-risk and high-risk groups among patients in terms of OS, T stage, N stage, M stage, clinical stage, and gender.

Using violin plots, we demonstrated the differences in risk scores among patients grouped by age, gender, M stage, clinical stage, and T stage (**Figure 5**). We noticed that the risk scores were significantly higher among older, female, stage III-IV, and T3-4 patients compared to younger, male, stage I-II, and T1-2 patients. These results suggest that the ICDRS is associated with poor prognosis in BLCA patients. **Figure 5** reveals the proportion of T stage distribution within the ICDRS risk subgroups, highlighting the differences in early versus late stages among patients at different risk levels.

Further survival analyses demonstrated the stable performance of the ICDRS across age and stage stratifications. As shown in **Figure 5**, KM curves reveal the survival probabilities of low-risk and high-risk groups as defined by the ICDRS among patients aged ≥60 and <60, as well as those in clinical stages I and II versus III and IV. In all subgroups, the high-risk group exhibited poorer survival rates compared to the low-risk group, and these differences were statistically significant (p < 0.05).

These results indicate that the ICDRS can effectively differentiate prognostic risks among BLCA patients with varying clinical characteristics, providing robust clinical decision support for personalized treatment.

### 3.5 Establishment and validation of a nomogram integrating clinical features

**Figure 6:**
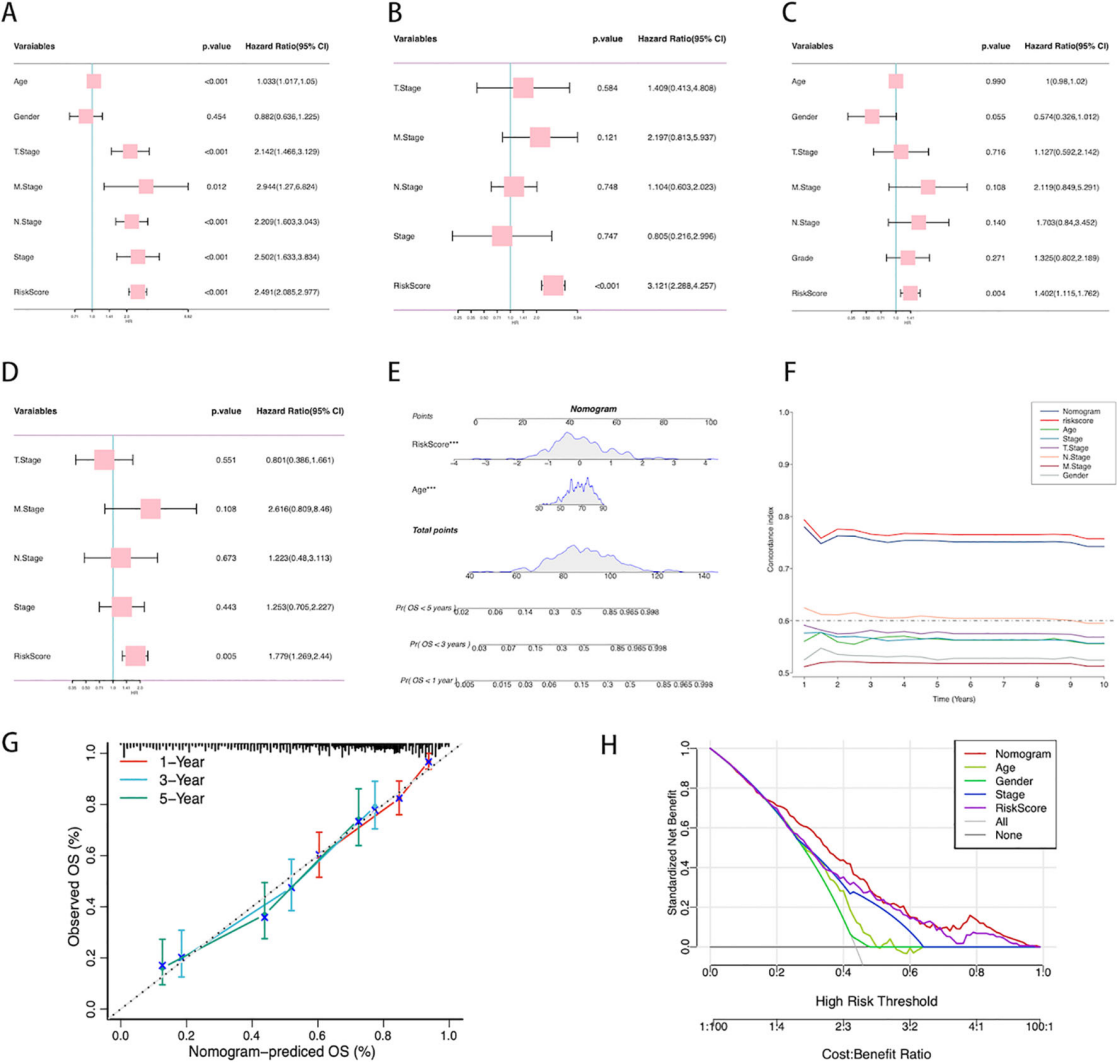
Establishment of the nomogram. **(A)** Univariate analysis of the clinical characteristics and ICDRS for OS in the TCGA-BLCA cohort. **(B)** Multivariate analysis in the TCGA-BLCA cohort. **(C)** Univariate analysis of the clinical characteristics and ICDRS for OS in the GSE13507 cohort. **(D)** Multivariate analysis in the GSE13507 cohort. **(E)** Construction of the nomogram based on ICDRS and clinical characteristics. Each variable’s contribution to the predictive model is represented by a point-line plot next to it, where a larger contribution indicates a stronger association with survival prediction. **(F)** Comparison of the C-index between the nomogram and other clinical characteristics. **(G)** Calibration curve of the nomogram for 1, 3, and 5-year OS. **(H)** Decision curve analysis showing the standardized net benefit of applying the nomogram compared to other clinical characteristics.

To evaluate whether the ICDRS is an independent prognostic factor for BLCA, we assessed the impact of age, gender, TNM staging, clinical staging, and ICDRS on patient OS in two cohorts: TCGA-BLCA and GSE13507. Univariate analysis results (**Figure 6C**) showed that in the TCGA-BLCA cohort, age, TNM staging, clinical staging, and ICDRS were all significant prognostic factors for OS. However, in the GSE13507 cohort, only ICDRS significantly impacted OS as an independent prognostic factor. Subsequent multivariate analyses (**Figure 6D**) further confirmed ICDRS as an independent prognostic indicator in both cohorts. In this analysis, ICDRS significantly influenced OS even after adjusting for other clinical features.

A prognostic scoring nomogram was constructed based on ICDRS and clinical features (**Figure 6**), integrating age and ICDRS scores. The nomogram’s predictions for 1-year, 3-year, and 5-year OS were highly consistent with the actual observations, as shown by the calibration curve (**Figure 6**). We also compared the nomogram’s C-index with that of other individual clinical features (**Figure 6**), and the results showed that its predictive capability for OS was superior to that of individual clinical features alone. Decision curve analysis (**Figure 6**) indicated that, within a certain range of high-risk thresholds, using the nomogram could achieve a higher standardized net benefit compared to other clinical features. This means that decision-making based on the nomogram offers superior expected benefits over traditional clinical feature-based decisions.

### 3.6 Transcriptomic feature analysis of different ICDRS patient groups

To further investigate the molecular mechanisms underlying the correlation between ICDRS and prognosis in BLCA, we conducted GSEA and GSVA. These analyses can reveal differences in biological processes and pathway activities related to patient groups with high and low ICDRS scores.

**Figure 7:**
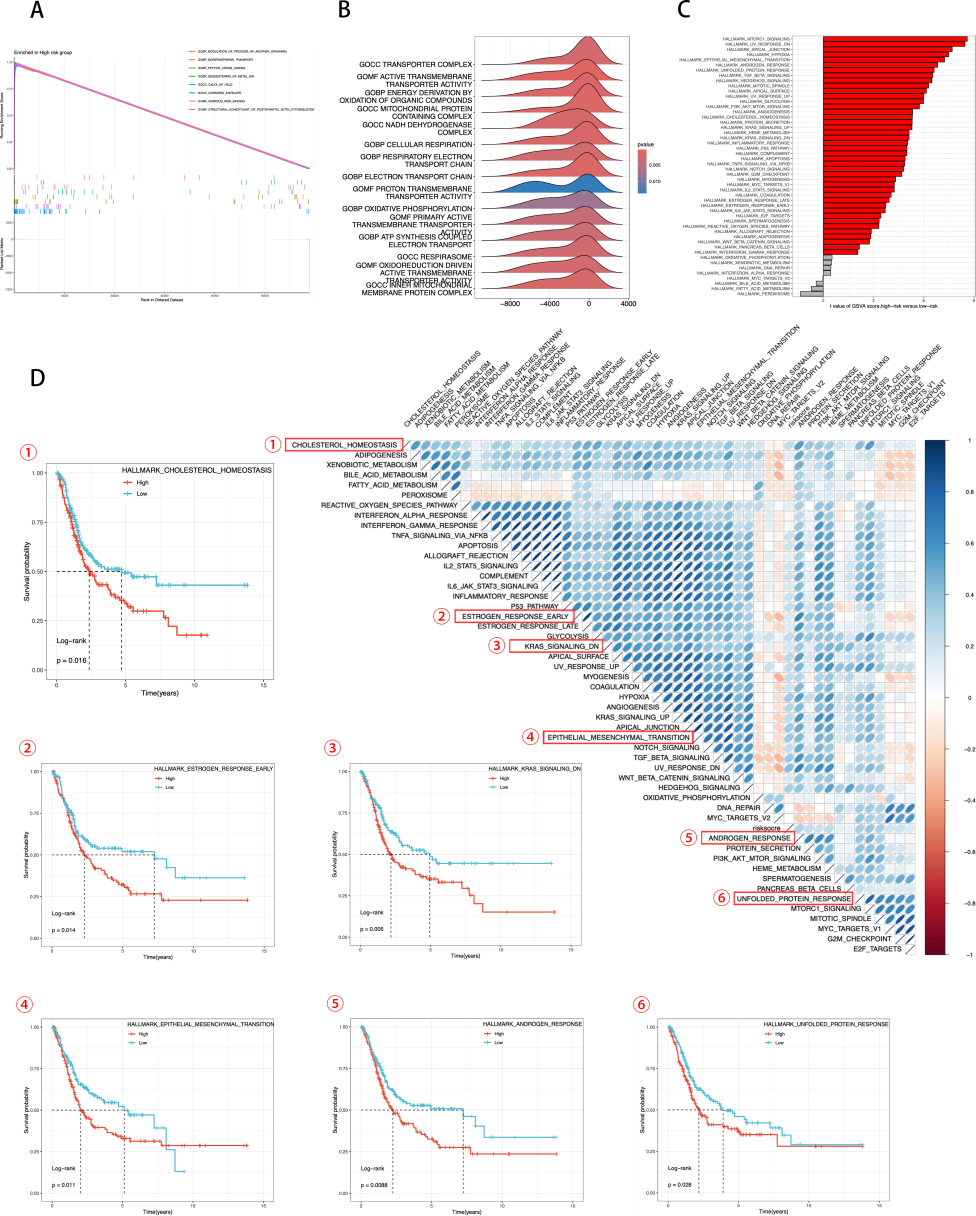
Transcriptomic features of various ICDRS patient groups. **(A, B)** GO terms enriched by GSEA analysis. **(C)** Differences in KEGG analysis between the high- and low-risk groups scored by GSVA. **(D)** Correlation between the risk score and hallmark pathway activities scored by GSVA. KM survival plots showing the significant correlations between the OS and GSVA scores.

**Figure 7** from the GSEA analysis reveals the GO pathways enriched in different ICDRS groups. **Figure 7** shows pathways related to structural constituent of postsynaptic actin cytoskeleton, norepinephrine transport, peptide cross-linking, sequestering of metal ion, calyx of held, cornified envelope, ferrous iron binding, and modulation of processes of another organism. **Figure 7B**’s ridge plot highlights enriched GO pathways that involve different aspects of TME. **Figure 7** provides GSVA scores for KEGG pathways, further enhancing our understanding of the pathway activities associated with ICDRS scores. Through KEGG pathway analysis, we can quantitatively compare differences in metabolism and signaling between groups with high and low ICDRS scores.

In **Figure 7**, using the Hallmark gene set, we evaluated GSVA scores to assess the correlation between ICDRS scores and activity of typical pathways, and through KM survival plots, we demonstrated a significant correlation between OS and GSVA scores. Integrating these data, we conclude that ICDRS scores are positively correlated with the activity of several key biological pathways, including cholesterol homeostasis, estrogen response, epithelial-mesenchymal transition (EMT), androgen response, and the unfolded protein response (UPR). Moreover, the upregulation of these pathways is significantly associated with poor prognosis in BLCA patients. Conversely, pathways negatively correlated with ICDRS, such as KRAS signaling, are associated with a better prognosis. These results suggest that the activation or inhibition of these pathways may contribute to the different prognostic outcomes observed within ICDRS subgroups. These findings underscore the importance of ICDRS as a potential prognostic biomarker in BLCA treatment and provide a scientific basis for future development of targeted therapies against these pathways.

### 3.7 Establishment and validation of an ICD-related gene risk scoring model based on Lasso regression

**Figure 8:**
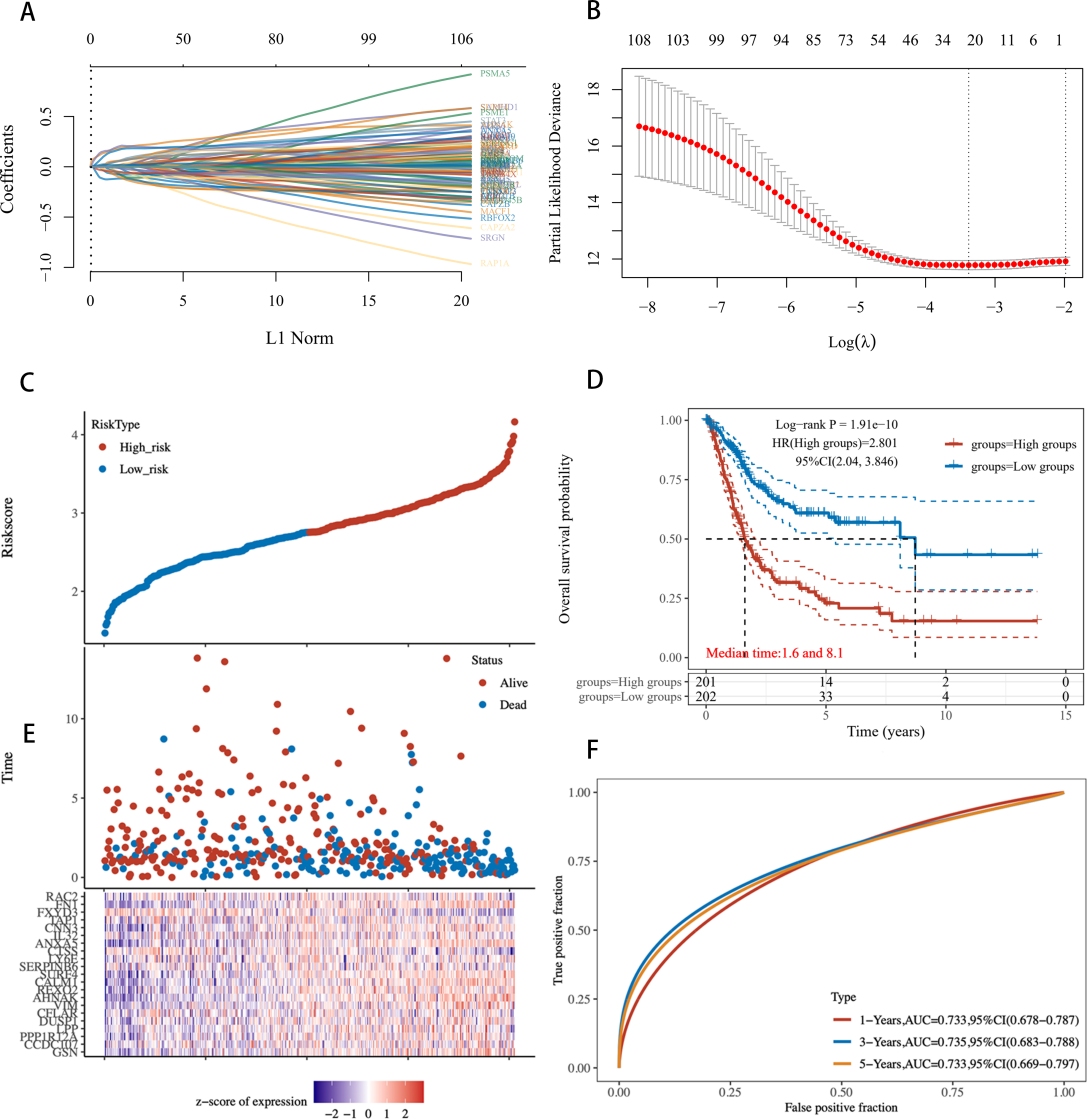
Risk scoring model based on 108 ICD-related genes constructed using Lasso regression. **(A)** Lasso Regression Coefficient Path Plot for 108 Genes. **(B)** Deviation in cross-validation for Lasso regression. The X-axis represents the logarithm of λ values, and the Y-axis represents deviation. Red points indicate the average deviation at each λ value, gray lines represent the standard error of the deviation, and the vertical line on the X-axis marks the optimal λ value. **(C)** Risk profile in the training set. **(D)** KM survival curves for high-risk and low-risk groups in the training set. **(E)** Distribution of Risk scores for each sample. **(F)** ROC curves for the training set.

Building on the previous results, given the robustness and universality of the Lasso-Cox model across most models, we revisited the modeling of all 108 genes using the Lasso-Cox approach. The Lasso regression coefficient path plot displayed how the coefficients of the 108 genes shrink towards zero as the L1 regularization penalty (λ value) increases, revealing the model variables ultimately selected (**Figure 8**). In Lasso regression, the cross-validation deviation plot identified the optimal λ value that minimizes the cross-validation error, providing the best model complexity (**Figure 8**). **Figure 8** shows the distribution of risk scores between high-risk and low-risk samples in the training set, calculated based on the selected λ value. The KM survival curves illustrate significant differences in prognosis between the high-risk and low-risk groups (P < 0.001), with median survival times of 1.6 years and 8.1 years, respectively (**Figure 8**). The risk score distribution plot shows the relationship between each sample’s score and survival status (alive/dead) in the training set, with high-risk scores associated with the occurrence of death events (**Figure 8**). The ROC curve evaluated the accuracy of the risk scoring model in predicting survival at 1 year, 3 years, and 5 years. The AUC values for 1-, 3-, and 5- year OS are 0.733, 0.735, and 0.733. The AUC at different time points showed the model’s strong predictive performance (**Figure 8**). These results indicate that the further developed ICDRS not only accurately distinguishes between high-risk and low-risk BLCA patients with powerful prognostic prediction capabilities but also significantly enhances the model’s robustness.

### 3.8 Comprehensive correlation analysis of ICDRS with single-cell characteristics

**Figure 9:**
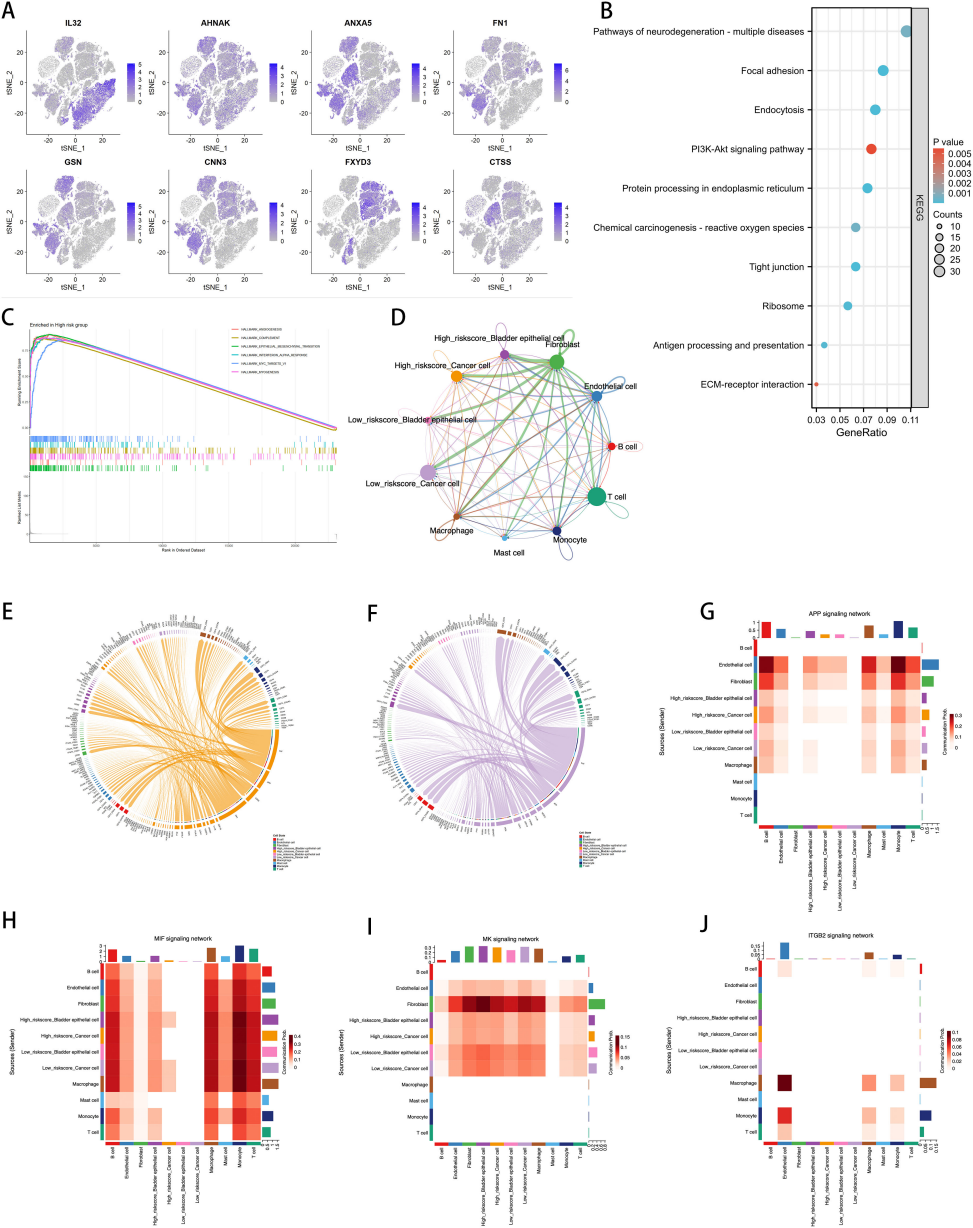
Correlation of ICDRS with Single-Cell Characteristics. **(A)** Expression of the 8 genes in various cell types by single-cell RNA-seq analysis. **(B)** KEGG analysis of the DEGs between the high- and low-risk cells. **(C)** GSEA analysis identified the GO terms enriched in the high-risk cells. **(D)** Circos plots showing the signaling pathway networks. **(E)** Ligand-receptor interactions sent from high-risk tumor cells. **(F)** Ligand-receptor interactions sent from low-risk tumor cells. **(G–J)** The heatmaps showing the roles of different cell types in the APP, MIF, MK, and ITGB2 signaling networks.

We intersected the top 10 upregulated and downregulated genes in the Ridge model with the high-weight genes from the LASSO analysis to identify the eight most prominent genes in the ICDRS, namely IL32, AHNAK, ANXA5, FN1, GSN, CNN3, FXYD3, and CTSS. We conducted a detailed analysis of the expression of ICDRS in different single-cell types and its functional associations (**Figure 9**). Through single-cell RNA sequencing analysis (**Figure 9**), we identified the expression patterns of these eight genes across various cell types, showing that these genes are primarily expressed in bladder epithelial cells, macrophages, monocytes, cancer cells, fibroblasts, and T cells. This analysis revealed the association of ICDRS with the functions of specific cell populations.

KEGG pathway analysis further identified functionally enriched pathways in DEGs between high-risk and low-risk cells (**Figure 9**). Results show that various biological processes and signaling pathways such as neurodegenerative disease pathways, the PI3K-Akt signaling pathway, and extracellular matrix (ECM)-receptor interactions are significantly enriched in high-risk cell populations. Through GSEA analysis, we identified enriched HALLMARK pathways in high-risk cells, including angiogenesis, activation of the complement system, EMT, interferon-α response, activation of MYC targets, and myogenesis, revealing the key biological processes these cells may be involved in (**Figure 9**).

The Cellchat diagram provides a detailed view of interactions among different cell types within specific signaling pathway networks (**Figure 9**), revealing the complexity and diversity of cell communication. Furthermore, we visualized the molecular interactions between tumor cells and other cell types with high and low risk scores. **Figures 9**, **9F** respectively show the ligand-receptor interactions observed in high-risk and low-risk tumor cells. Compared to the low-risk group, the high-risk group displays specificity in the FN1 and CD99 signaling pathways. FN1, a major extracellular matrix protein, is involved in various cellular processes, including cell adhesion, migration, wound healing, and embryonic development. Increased expression of FN1 is often associated with higher malignancy, poor prognosis, and enhanced invasiveness of cancer. The active FN1 signaling in the high-risk group may indicate these cells have greater invasive capabilities and metastatic potential. CD99, a cell surface glycoprotein widely expressed in many cell types, is involved in cell adhesion, migration, and immune regulation. In high-risk tumor cells, the expression of CD99 may be associated with immune evasion, cell migration, and invasion.

To further discuss the expression and correlation of pathways across various cell types, we selected several pathways for further heatmap visualization, including the APP pathway, which influences cell proliferation and death, the MIF pathway associated with the worsening prognosis of various cancers, and the MK and ITGB2 pathways, which are linked to tumor aggressiveness and poor prognosis (**Figure 9**). The results indicate that in high-risk tumor cells, the APP, MIF, and MK pathways exhibited more significant expression compared to the low-risk group. Moreover, the MIF pathway showed a high correlation with fibroblasts, corroborating the specific expression of FN1 as seen in **Figure 9**. However, the ITGB2 pathway did not show differences in expression in BLCA and had very low expression levels, suggesting that this pathway is less relevant to BLCA. These heatmaps provide an intuitive view for understanding the role of ICDRS across different cell types and signaling pathways, supporting the importance of ICDRS in cell communication and functional execution within TME.

### 3.9 Immune landscape associated with ICDRS in BLCA

**Figure 10:**
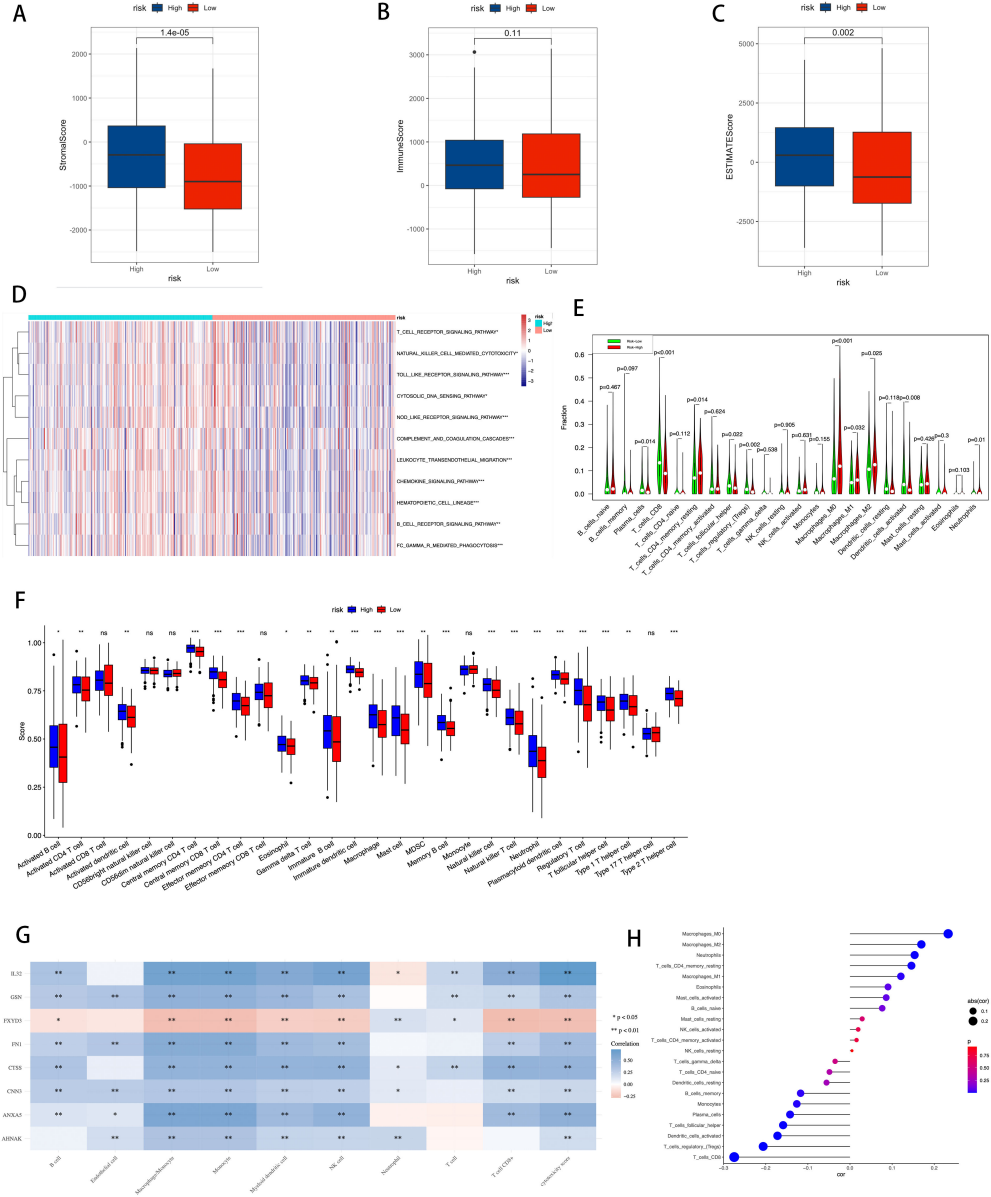
Correlation between TME, immune characteristics, and ICDRS. **(A–C)** The Stromal Score, Immune Score, and ESTIMATE Score were used to quantify the different immune statuses between the high- and low-risk groups. **(D)** Activity of immune-related pathways showed significant differences between the high- and low-risk groups. **(E)** Using the CIBERSORT algorithm to calculate the abundance of each type of TME-infiltrating cell in the high and low-risk groups. **(F)** Quantitative scoring of infiltrating cell abundance using the scheme for 28 types of immune phenotype scores. **(G)** The association between TME-infiltrated cells and genes built into the ICDRS. **(H)** Correlation analysis between TME-infiltrated cells and the ICDRS. Statistical significance is indicated as follows: * for p-value < 0.05, ** for p-value < 0.01, *** for p-value < 0.001, and ns (not significant) for p-value ≥ 0.05.

**Figure 10** provides a detailed display of the differences in the immune landscape between high-risk and low-risk groups in BLCA patients. Using the Stromal Score, Immune Score, and ESTIMATE Score, we quantified the varying immune states between the two risk groups (**Figure 10**), revealing that the high-risk group has higher Stromal Scores (P = 1.4e-5), Immune Scores (P = 0.11), and ESTIMATE Scores (P = 0.002).

Additionally, using the ssGSEA algorithm, differences in the activity of immune-related pathways were confirmed between the high-risk and low-risk groups, including T cell receptor signaling pathway, natural killer cell mediated cytotoxicity, Toll-like receptor signaling pathway, cytosolic DNA sensing pathway, NOD-like receptor signaling pathway, complement and coagulation cascades, leukocyte transendothelial migration, chemokine signaling pathway, hematopoietic cell lineage, B cell receptor signaling pathway, and FC gamma R mediated phagocytosis (**Figure 10**). This demonstrates that the activation status of these pathways varies in the TME of different risk levels. To further analyze the differences in specific immune cell infiltration between the high-risk and low-risk groups, we used the CIBERSORT algorithm to calculate the abundance of each TME infiltrating cell type in high-risk and low-risk groups (**Figure 10**). It was found that high-risk groups had a higher abundance of T cells CD4 memory resting, macrophages M0, macrophages M1, and macrophages M2, while the low-risk group had a higher abundance of T cells CD8, T cell follicular helper, T cells regulatory, and dendritic cells activated. Subsequently, these findings were validated using a quantitative scoring scheme for 28 types of immune cell phenotypes (**Figure 10**) and an analysis of the correlation between immune cells and risk scores (**Figure 10**), both yielding consistent results.

The study further investigated the correlation between infiltrating cells in the TME and the eight genes constituting the ICDRS (**Figure 10**), revealing associations between specific immune cell subpopulations and gene expression patterns in the ICDRS.

These findings collectively point to the ICDRS as an effective tool for quantifying the immune status of BLCA patients, indicating significant differences in the immune landscape characteristics of patients at different risk levels.

### 3.10 Drug sensitivity prediction and HPA validation

**Figure 11:**
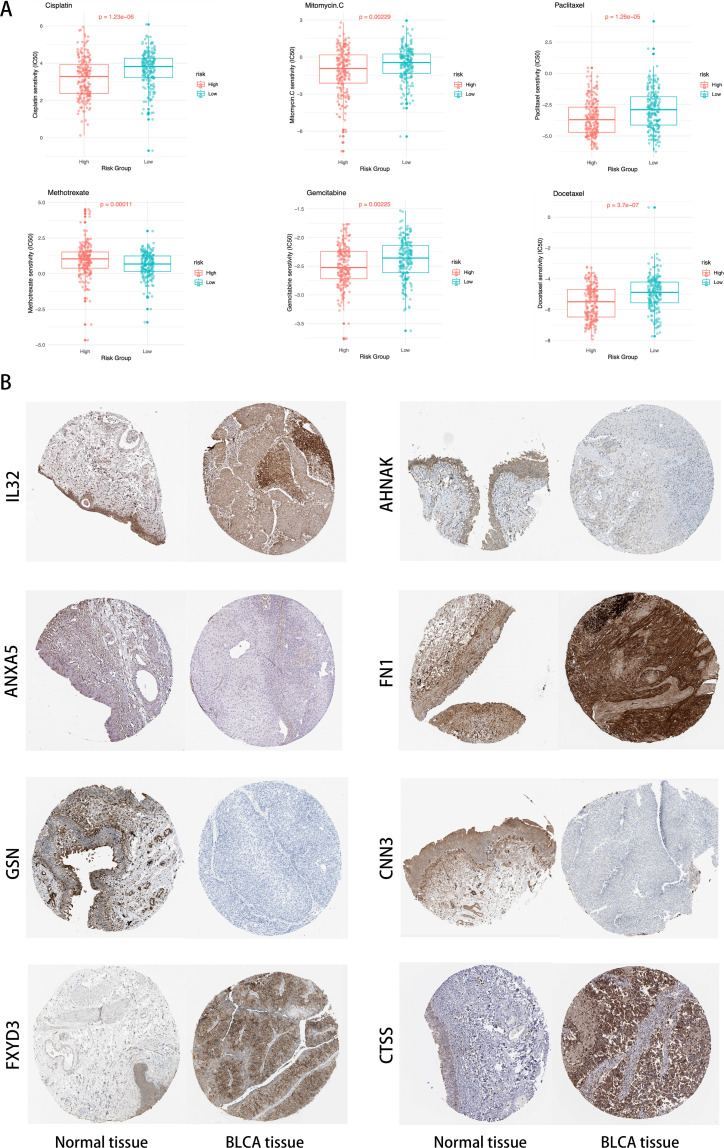
**(A)** Distribution of IC50 scores for drugs in high- and low-risk groups defined by ICDRS. **(B)** Validation of the eight genes expression in the HPA database.

By analyzing the GDSC database, we calculated the IC50 values for commonly used drugs in the treatment of BLCA across different cancer cell lines. Specifically, significant differences were observed in the IC50 values for Cisplatin, Mitomycin C, Paclitaxel, Methotrexate, Gemcitabine, and Docetaxel between different risk groups (**Figure 11**). This highlights the potential value of these gene expression levels in predicting BLCA patients’ responses to specific chemotherapy drugs.

In **Figure 11**, compared to normal tissue, IL32, FN1, FXYD3, and CTSS are significantly upregulated in BLCA tissues, ANXA5, AHNAK, GSN, and CNN3 are significantly downregulated in BLCA tissues.

## 4 Discussion

In the field of BLCA treatment, the current reliance on costly invasive surgeries makes it one of the most expensive cancers to treat (44). Therefore, exploring new methods to reduce treatment costs is particularly important. In the new era of immunotherapy, methods that classify, intervene, and predict cancer based on immune characteristics are increasingly becoming a research focus (45). Leveraging advancements in modern genomics and transcriptomics, this study uses single-cell analysis methods to calculate ICD scores and explore DEGs. Using the WGCNA method, gene clusters were constructed in TCGA database samples, and after integrating single-cell and TCGA data, key genes related to ICD scores were identified. These genes were then simulated through hundreds of machine learning models to predict patient long-term survival, and the predictive performance of the models was validated using external datasets. Additionally, the effectiveness and robustness of the predictive model were further confirmed through clinical data. To understand the functions of key genes in the model, GSEA and GSVA were conducted to explore related biological function pathways, and the survival impacts of these pathways were analyzed, thus affirming the importance of the new predictive model. Finally, by testing the model in multiple immune databases and scoring systems, and combining it with drug sensitivity analysis, we assessed the variability of drug therapy in model predictions, laying the foundation for further clinical application and expansion of the model.

In this study, we integrated multi-omics datasets, including single-cell and bulk data, to enhance the comprehensiveness and depth of our analysis. We divided the TCGA database into a training set and an internal testing set, adding an external testing set to validate the generalizability of the model. Notably, the internal testing set was not used in the model training process. In the comparison of various machine learning models, although the RSF model showed extremely high accuracy on the training set, its accuracy significantly decreased on the testing set, indicating potential overfitting issues. To overcome this, we focused particularly on Ridge and Lasso regression models, combining the fitting results of a hundred machine learning models. Both models employ regularization techniques to reduce the risk of overfitting but differ in how they handle variable selection and complexity adjustment. The Ridge model applies L2 regularization to shrink all variables, suitable for dealing with highly correlated variables. In contrast, the Lasso model employs L1 regularization to achieve sparse selection of variables, compressing the coefficients of unimportant variables to zero, thus demonstrating advantages in model simplification and variable selection. In our study, the Ridge model was deemed more suitable due to its advantage in maintaining variable stability. Additionally, we explored the combination of the Lasso model with the Cox proportional hazards model, particularly important in survival analysis, allowing for deeper exploration and validation of biomarkers and frequently appearing in other studies. These analyses showed that the combination of Lasso and Cox models not only provided results similar to other studies but also enhanced the predictive accuracy and interpretability of the model on clinical data. Through precise model selection and algorithm comparison, our research not only improved the accuracy of predictive models but also provided new molecular targets for future BLCA treatment strategies.

Initially, we evaluated ICD features using single-cell transcriptomic analysis methods. The results showed that immune cells such as macrophages, T cells, and monocytes exhibited high ICD activity, further confirming ICD’s critical role in stimulating anti-tumor immune responses. In contrast, non-immune cell types such as fibroblasts, endothelial cells, and cancer cells displayed lower ICD activity. Further analysis of the 108 ICDRgenes through GO and KEGG enrichment revealed that the GO analysis predominantly points to the regulation of the actin cytoskeleton pathway, closely related to the crucial role of the actin cytoskeleton in tumor cell migration and invasion in BLCA. This regulation not only affects the TME but may also impact the interactions between tumor cells and immune cells, potentially influencing immune responses. Research indicates that the occurrence of ICD depends not only on the immune cells’ recognition and elimination of tumor cells but also on the presentation of tumor cell surface antigens, which is closely linked to the regulation of the actin cytoskeleton (46–48). In the KEGG analysis, in addition to evidence related to actin cytoskeleton regulation, we found that ICDRgenes associated with viral infection pathways were enriched in BLCA, highlighting the potential for viral infections to promote tumor development through chronic inflammation or impacting immune surveillance (49, 50).

We conducted a comprehensive comparison between our model and previous gene prognostic models. Wang et al.’s risk score model based on methylation-driven genes achieved an AUC of 0.698 for 3-year OS in BLCA (51). Liang et al.’s model constructed from ferroptosis-associated genes demonstrated an AUC of 0.729 for 5-year OS (52). In contrast, our model achieved AUC values of 0.733, 0.735, and 0.733 for 1-year, 3-year, and 5-year OS, respectively. These results suggest that our ICDRS model offers superior predictive performance and greater stability across different survival timeframes compared to existing models, highlighting its potential value for clinical application in BLCA prognostication. In exploring the correlation between ICDRS and BLCA prognosis, our analysis of transcriptomes from different ICDRS risk subgroups uncovered significant differences in energy metabolism processes and TME-related pathways between groups with high and low ICDRS scores. GSVA scoring analysis using the Hallmark gene set further revealed significant associations between these scores and OS. Notably, ICDRS scores were found to positively correlate with the activation of several key biological pathways, including cholesterol homeostasis, early estrogen response, EMT, androgen response, and UPR, all of which are linked to poor prognosis. Cholesterol homeostasis is crucial for maintaining tumor cell membrane integrity, producing bioactive molecules, and providing energy, while disturbances in cholesterol metabolism may enhance tumor invasiveness and metastasis (53, 54). This finding aligns with recent studies in BLCA, where Liang et al. demonstrated that targeting cholesterol metabolism inhibited BLCA proliferation (55). Early estrogen responses might accelerate BLCA cell proliferation and progression by activating specific estrogen receptor pathways (56, 57). EMT facilitates tumor metastasis by reducing intercellular adhesion and increasing migratory and invasive capacities (58). Dysregulation of EMT has proven to drive the progression of BLCA (59–61). The androgen response impacts tumor biology by regulating the cell cycle and apoptosis (62). The androgen response pathway has particular relevance in BLCA as recent studies have revealed sex disparities in outcomes, with Chen et al. and Li et al. demonstrating that androgen receptor expression correlates with advanced stage and poor prognosis specifically in male BLCA patients (63, 64). And the enhanced UPR helps tumor cells survive under adverse conditions like hypoxia and nutrient deficiency (65, 66). In contrast, the KRAS signaling pathway’s activity inversely correlates with ICDRS and aligns with a better prognosis. The activation of KRAS may decrease tumor cells’ adaptability to treatments and promote apoptosis, while its upregulation might inhibit tumor-promoting pathways and enhance immune surveillance, thereby preventing tumor escape (67). Overall, the KRAS pathway potentially exerts a positive effect on BLCA prognosis by orchestrating tumor growth regulation and immune response, highlighting its role in tumor biology and as a target for therapeutic intervention.

By integrating single-cell transcriptomic data, we further unveiled the molecular mechanisms associated with ICDRS. Our ICDRS comprises eight key genes (IL32, AHNAK, ANXA5, FN1, GSN, CNN3, FXYD3, CTSS) with diverse biological functions in the tumor microenvironment. CTSS, ANXA5, GSN, AHNAK and IL-32 have established roles in ICD: CTSS inhibition has non-redundant therapeutic potential to enhance anti-tumor immune responses (68). ANXA5 acts as an immunostimulatory agent to render apoptotic tumor cells immunogenic and induce tumor regression (69, 70). Galluzzi et al. proved that cells can avoid ICD by secreting large amounts of GSN (8). IL-32 has the metastasis-promoting effect in BLCA (71). And FXYD3 is an unfavorable prognostic biomarker associated with hypoxia, pro-tumor TILs, and T cell exhaustion (72). FN1 and CNN3 are genes encoding extracellular matrix and cytoskeleton-related proteins (73). Analysis revealed that eight characteristic genes were primarily expressed in bladder epithelial cells, macrophages, monocytes, cancer cells, fibroblasts, and T cells, illustrating the complex roles of ICDRS in tumor development and immune regulation. Specifically, gene expressions in bladder epithelial cells and cancer cells may be directly related to tumor oncogenesis. M1 macrophages exhibit high anti-tumor activity, whereas M2 macrophages may suppress immune responses and support tumor growth and metastasis (74). Monocytes are crucial for initiating and maintaining anti-tumor immunity, while TME can alter their differentiation and function, sometimes promoting tumor survival and immune evasion (75, 76). Fibroblasts play a significant role in tumor fibrosis, intercellular signaling, and maintaining tumor structure (77, 78). T cells can directly recognize and kill tumor cells or enhance the attack against tumors (79). Additionally, immune cell infiltration analysis showed that in the high ICDRS group, cell types such as T cells CD4 memory resting and various macrophages were more abundant, whereas the low ICDRS group was enriched with activated immune effector cells like T cells CD8, T cell follicular helper, T cells regulatory, and activated dendritic cells.

ICD and ICDRS scores are closely related, and while tumors with high ICDRS scores would theoretically exhibit strong immune cell infiltration, this does not necessarily imply effective immune-mediated tumor control. In fact, tumors with high ICDRS may evade immune surveillance by promoting an immunosuppressive microenvironment, reflecting a poorer prognosis. For example, the enrichment of M2 macrophages creates an immunosuppressive environment that promotes chemotherapy resistance by enhancing tumor cell survival and DNA damage repair mechanisms, with previous studies showing that M2 macrophage abundance is significantly positively correlated with BLCA progression and metastasis (80, 81). Cell-cell interaction analysis demonstrated reduced effector T cell-tumor cell engagement via diminished co-stimulatory signaling and enhanced inhibitory checkpoint interactions in high ICDRS tumors, providing a mechanistic basis for immune evasion despite the presence of immune cells. In contrast, tumors with low ICDRS scores may be more likely to stimulate effective cytotoxic T cell responses, suggesting a more favorable immune environment and better prognosis. These findings indicate that the specific conditions of the TME in BLCA play a crucial role in determining the prognostic value of ICDRS, pointing to the need for more personalized treatment strategies for BLCA patients with high ICDRS scores to optimize their prognosis. In summary, these research results not only provide new insights into the mechanisms of BLCA progression but also offer a scientific basis for developing more targeted immune-mediated treatment strategies.

The potential clinical application of ICDRS in BLCA management could significantly enhance current treatment. At initial diagnosis, ICDRS could complement conventional risk stratification, potentially identifying high-risk patients who might benefit from earlier aggressive intervention or patients requiring intensified neoadjuvant approaches. Our drug sensitivity findings suggest ICDRS could guide therapy selection, directing high-risk patients (showing resistance to conventional chemotherapeutics) toward alternative treatments like immunotherapy or targeted agents. Additionally, ICDRS could serve as a biomarker for monitoring treatment response, with changes during therapy potentially indicating resistance development. From an implementation perspective, the eight-gene signature could be assessed using RT-PCR or targeted RNA sequencing, making it feasible for integration into clinical testing workflows alongside established risk factors to guide treatment decisions throughout the BLCA care continuum.

Although this study utilized multi-omics datasets for a comprehensive analysis and explored various aspects such as clinical features, immune infiltration, and TME, there are still significant limitations to address. Firstly, despite our attempts to validate the model using external datasets, batch effects between datasets and differences in gene numbers led to significant heterogeneity across datasets. This heterogeneity not only complicates model validation but may also impact the consistency and accuracy of the model across different datasets. Additionally, since the samples might predominantly originate from specific clinical settings or geographic locations, this limits the broad applicability and generalizability of the study results. Secondly, in terms of technical choices, although Lasso and Ridge regularization models were used to reduce overfitting, these models still have limited capabilities in handling extreme data and capturing non-linear relationships. Furthermore, the selection of regularization strength is a challenge in itself and could introduce additional biases. Although we conducted gene function enrichment and pathway analysis, these analyses might not be sufficient to fully reveal the complex biological mechanisms and pathways involved. From a clinical application perspective, although our model has been statistically validated for effectiveness, its real-world application might face several challenges such as patient acceptance, treatment costs, and operational complexity. Additionally, while drug sensitivity prediction offers potential for personalized treatment, the impact of experimental conditions, drug dosages, and individual biological variations may cause fluctuations in prediction outcomes.

In summary, future efforts should focus on enhancing the generalizability of the model by incorporating samples from a broader range of ethnicities and regions to verify the model’s applicability. Additionally, further research should aim to integrate more data at the biological level to strengthen the model’s biological interpretability and to explore the challenges and solutions that may arise in actual clinical applications. These efforts will help to increase the practical impact and scientific value of the research.

## Data Availability

The original contributions presented in the study are included in the article/supplementary material. Further inquiries can be directed to the corresponding authors.
